# Duration of progesterone exposure before frozen embryo transfer impacts live birth rates following single vitrified-thawed day 6 blastocyst transfer: a multicenter cohort study

**DOI:** 10.1186/s40834-026-00425-3

**Published:** 2026-01-28

**Authors:** He Cai, Zhiqiang Wang, Ying Fang, Zan Shi, Danmeng Liu, Xiaokui Yang, Yali Ni, Juanzi Shi

**Affiliations:** 1 Assisted Reproduction Center, Northwest Women’s and Children’s Hospital, 73# Houzaimen North Street, Xi’an, 710000 China; 2 Reproductive Medical Center, Gansu Provincial Maternity and Child-care Hospital, Gansu, China; 3 Department of Human Reproductive Medicine, Beijing Obstetrics and Gynecology Hospital, Beijing, China; 4 Translational Medicine Center, Northwest Women’s and Children’s Hospital, Xi’an, China

**Keywords:** Progesterone exposure, Frozen embryo transfer, Vitrified day 6 blastocyst, Live birth, Slow developing, Hormone replacement therapy

## Abstract

**Research question:**

Does administering progesterone for 6 days prior to frozen embryo transfer (FET) improve live birth rate (LBR) compared to 5 days of progesterone exposure when transferring blastocysts expanded on Day 6 (D6)?

**Methods:**

This multicenter retrospective cohort study included 1,639 single FET cycles using D6 blastocysts under hormone replacement therapy (HRT) between January 2018 and December 2023. Cycles were stratified by duration of progesterone priming: 6 days (P6 group, *n* = 1,122) or 5 days (P5 group, *n* = 517). The primary outcome was LBR; secondary outcomes included biochemical and clinical pregnancy rates, miscarriage rate, and neonatal outcomes (birth weight and gestational age at delivery). Generalized estimating equation (GEE) regression models were used to adjust for potential confounders. Subgroup analyses by PGT status and sensitivity analyses restricted to first FET cycles or good-quality blastocysts were also conducted.

**Results:**

The P6 group demonstrated a significantly higher LBR than the P5 group (33.87% [380/1,122] vs. 24.95% [129/517]; *P* < 0.001). After multivariable adjustment, the P6 regimen remained independently associated with increased LBR (adjusted odds ratio [aOR] 1.72; 95% CI 1.32–2.19; *P* < 0.001). Biochemical pregnancy (51.96% vs. 37.72%; aOR 2.10; 95% CI 1.64–2.62) and clinical pregnancy rates (46.52% vs. 33.66%; aOR 1.94; 95% CI 1.50–2.47) were also significantly higher with 6 days of progesterone priming. No significant differences were observed in miscarriage rates or neonatal outcomes between groups. Sensitivity analyses including restriction to first FET cycles, good-quality blastocysts, or stratification by blastocyst ploidy status did not alter the primary findings.

**Conclusion:**

Extending progesterone exposure to 6 days before FET is associated with a significantly higher live birth rate compared with a 5-day protocol in transfers of vitrified–thawed Day 6 blastocysts. These findings warrant confirmation in prospective, large-scale randomized controlled trials.

**Clinical trial number:**

Not applicable

**Supplementary Information:**

The online version contains supplementary material available at 10.1186/s40834-026-00425-3.

## Introduction

The use of frozen embryo transfer (FET) in ART has increased dramatically over the past decade and continues to rise globally [1]. Endometrial preparation for FET can be achieved via natural or modified natural cycle, mild stimulation or hormone replacement therapy (HRT), with sequential estrogen and progesterone administration. While no definitively superior protocol for endometrial preparation in FET cycles has been proven [2], HRT seems to be associated with reduced live birth rates, increased miscarriage risk, and adverse obstetric/neonatal outcomes compared to natural cycles [3, 4]. Despite this, HRT remains an integral part of everyday clinical practice, due to its minimal cycle monitoring, lower cost and greater scheduling flexibility [5, 6].

Given the popularity of FET and growing concerns regarding HRT for endometrial preparation, there is a continued need for research to refine the protocol and improve clinical outcomes [7, 8]. The timing of progesterone start is critical in HRT-FET cycles, as it triggers the secretory transformation of the endometrium and serves as a key temporal regulator of the window of implantation, determining its opening and closure.

In clinical practice, the duration of progesterone exposure prior to FET is typically based on embryonic developmental stage: 5 days of progesterone administration for blastocyst-stage embryos and 4 days for cleavage-stage embryos cultured overnight to Day 4 [8, 9]. However, it remains unclear whether this protocol applies to blastocysts developing on Day 6 (D6), which are empirically transferred after the same duration of progesterone exposure as Day 5 (D5) blastocysts in HRT cycles [10]. Notably, a retrospective study by Roelens et al. [11]. challenged the uniform application progesterone priming, suggesting that the timing of progesterone starting in HRT cycles may require adjustment according to the embryo’s developmental stage. In their subgroup analysis on D6 blastocysts, a non-significant trend toward higher live birth rates (LBR) was observed with 6 days of progesterone exposure compared to 5 days (35.5% vs. 21.5%). Though underpowered for statistical significance, these findings suggest the hypothesis that extended progesterone priming may improve endometrial-embryonic synchrony in slower-developing embryos.

Given the limited data on the optimal duration of progesterone administration before FET for slow-growing D6 blastocysts, we evaluated whether extending progesterone exposure to 6 days improves ART outcomes compared to the 5-day regimen in a large cohort undergoing single vitrified-thawed D6 blastocyst transfers.

## Materials and methods

### Study design

We conducted a retrospective cohort study that included all single autologous vitrified–thawed D6 blastocyst transfers performed following HRT in three reproductive medicine centers in China (Northwest Women’s and Children’s hospital, Gansu Provincial Maternity and Child-care Hospital, and Beijing Obstetrics and Gynecology Hospital). Two cohort groups with vitrified–thawed D6 blastocysts were compared according to the duration of progesterone exposure before FET (6 days versus 5 days).

All data were collected retrospectively from the participating clinics’ electronic medical record databases and were deidentified. This study was approved by the Institutional Review Board at Northwest Women’s and Children’s Hospital (IRB#20250073).

### Patient population

All HRT-FET cycles with single D6 vitrified–thawed blastocyst transfer between January 2018 and December 2023 were screened. The warmed D6 blastocysts used in the study were from one of the following scenarios: supernumerary D6 blastocysts vitrified following selection of a top-grade embryo for fresh transfer or D6 blastocysts cryopreserved during freeze-all cycles. Inclusion criteria comprised endometrial preparation using HRT with or without GnRH agonist pretreatment. Exclusion criteria were: (1) inadequate endometrial thickness (< 6 mm); (2) FETs from vitrified oocytes or oocyte donation; (3) HRT cycles with spontaneous follicle growth or ovulation during endometrial preparation; (4) cycles with missing data regarding pregnancy outcomes.

### Clinical and laboratory procedures

Patients underwent ovarian stimulation according to standardized protocols determined by physicians across the three centers [12]. The most commonly used protocol was the GnRH antagonist protocol. Oocyte retrieval was performed 36 to 37 h later by transvaginal ultrasonographyeguided aspiration. The oocytes were inseminated 3 to 4 h later using conventional IVF or ICSI depending on the semen parameters and previous fertilization failure of IVF.

All participating clinics use − 5 series medium (Vitrolife, G€oteborg, Sweden). In brief, the zygotes were cultivated in G1–plus medium until they reached the cleavage-stage, and then transferred to G2–plus medium for further culture until they reached the blastocyst-stage (D5 or D6) [13]. Blastocyst morphology was evaluated based on the Gardner grading system prior to transfer [14]. The verification and warming procedures were executed in accordance with standardized protocols [15, 16]. Blastocysts were vitrified when reaching a Gardner grade better than 3CC (with a minimum morphology of 4CC, 3BC, or 3CB). Good-quality blastocysts were scored ≥ 3BB.

### Endometrial preparation and embryo transfer

In pure HRT cycle, estrogen replacement was administered via oral estradiol valerate (Progynova; Bayer, Berlin, Germany) and/or a transdermal estradiol gel (Oestrogel, BESINS, Belgium) which were continued for at least 10 days. Once the endometrial thickness achieved an adequate thickness as determined by the physician and the serum progesterone level was < 1.5ng/mL, exogenous progesterone was introduced. In cycles with gonadotropin-releasing hormone agonist (GnRH–a) combined with HRT, GnRH–a (3.75 mg; Diphereline, Ipsen Pty Ltd., France) was injected on day 2–5 of menstruation. Estrogen stimulation was used as in the HRT cycles in 28 to 30 days later [17]. Vitrified D6 blastocysts were thawed and transferred in the morning (10:00–13:00) after five (P5 group) or six (P6 group) days of progesterone exposure, with the day of progesterone initiation defined as P0. Study procedures including criteria for cycle cancellation were within standard of care for FET across sites, with some specifics such as the choice of estradiol and progesterone used, at the discretion of the treating physician. Progesterone was administered according to each participating center’s standard protocol, either as intramuscular progesterone in oil (60 mg once daily; Xianju Pharmaceutical, Zhejiang, China), vaginal Crinone^®^ (90 mg once daily; Merck Serono, Hertfordshire, UK), or oral Utrogestan^®^ (200 mg three times daily; Besins Healthcare, France). From the day of FET, all patients, irrespective of their initial progesterone regimen (vaginal or intramuscular), received adjunctive oral dydrogesterone (10 mg twice daily; Duphaston^®^, Abbott) as part of standardized luteal phase support across all three centers.

Embryo transfer was performed by senior gynecologists under transabdominal ultrasound guidance. Exogenous estrogen would be reduced after the confirmation of clinical pregnancy. Patients who achieved pregnancy continued the same progesterone regimen until 10 weeks of gestation (WG).

### Outcome measurements

The primary outcome was the LBR, defined as the delivery of any viable infant at 24 weeks or more of gestation [18]. Secondary outcomes included biochemical pregnancy (assessed by the serum β-hCG level 12–14 days after embryo transfer); Clinical pregnancy (defined as the presence of a visible gestational sac on ultrasound); miscarriages (defined as fetal loss before 20 WG), ectopic pregnancies (defined as a gestational sac located outside the uterine cavity); and stillbirth (intrauterine or intrapartum fetal death after 20 WG) [19].

### Statistical analysis

The frequency histograms and the Shapiro test were used to assess the normality of continuous variables. The baseline and cycle characteristics variables were presented as medians and interquartile ranges (IQR) for non–normal distributed continuous variables, compared using the Mann-Whitney U test. Categorical data were shown as counts and percentages, compared using χ^2^ tests or Fisher’s exact test.

Generalized estimating equation (GEE) models were used to compared outcomes between P6 versus P5 in D6 vitrified blastocyst transfer cycles, with patients as the random subject. In the GEE models, binomial distribution and logit link function were used for estimating odds ratios (OR) and the corresponding 95%CIs. Possible confounders selected based on the literature (female age, BMI, gravidity, PGT status [tested vs. untested], number of oocyte retrievals, number of blastocysts frozen, blastocyst origin (surplus from fresh cycle vs. freeze-all cycle), blastocyst quality [good vs. Fair], duration of estradiol priming, endometrial thickness, FET rank and progesterone used for luteal phase support) were included in the regression model [20–22]. Subgroup analyses by PGT status and sensitivity analyses restricted to either first FET cycles and good-quality blastocysts were also performed.

All analyses were conducted using the statistical packages R (v.3.4.3; The R Foundation) and *P*-values < 0.05 were considered statistically significant.

## Results

**Fig. 1:**
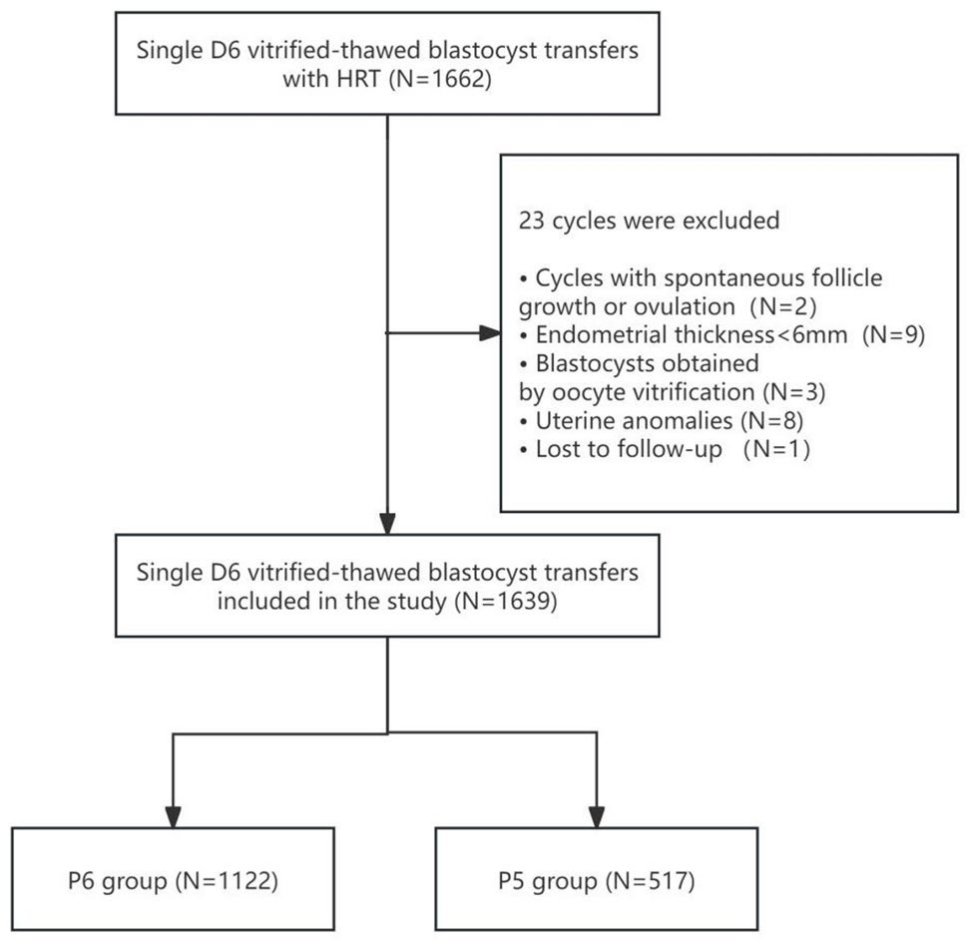
Flowchart of study cohort selection

Overall, 1,639 single D6 vitrified–thawed blastocyst transfers were analyzed in this study, with 1,122 in P6 group and 517 in the P5 group (Fig. 1). Baseline and cycle characteristics are presented in Table 1. The median female age (retrieval 32.0 and transfer: 33.0 years), male age (33.0 vs. 34.0 years), BMI (22.5 vs. 22.3 kg/m^2^) were comparable between the two groups. The distribution of infertility causes, insemination methods, rank of oocyte retrieval as well as the duration of estradiol treatment prior to progesterone initiation were also similar. Endometrial thickness was statistically significantly reduced in the P6 group compared to the P5 group (*P* = 0.001), despite identical medians of 10.0 mm (IQR 9.0–11.0), with a mean difference of only 0.3 mm. The P5 group had a higher oocyte yield (median 12 vs. 11, *P* < 0.001), a greater proportion of warmed blastocysts from freeze-all cycles (69.1% vs. 55.3%, *P* < 0.001), and a higher rate of first FET cycles (65.6% vs. 57.0%, *P* = 0.001). The utilization of PGT was also higher in the P5 group (22.44% vs. 17.20%, *P* = 0.012). No significant differences were found in the proportion of good-quality blastocysts (21.12% vs. 17.99%, *P* = 0.141), median number of blastocysts frozen (2.0 in both groups, *P* = 0.816) or progesterone for luteal support (32.17% vs. 36.56% received intramuscular and 67.83% vs. 63.44% received vaginal progesterone in the P6 and P5 groups, respectively, *P* = 0.081).

**Table 1:** Baseline demographics and cycle characteristics

Characteristics	Group P6 (*N* = 1122)	Group P5 (*N* = 517)	*P*–value
Female age of oocyte retrieval, median, IQR, y	32.0 (29.0–35.0)	32.0 (29.0–35.0)	0.898
< 30	346 (30.84%)	156 (30.17%)	0.697
30–34	486 (43.32%)	211 (40.81%)	
35–37	157 (13.99%)	82 (15.86%)	
38–40	97 (8.65%)	52 (10.06%)	
> 40	36 (3.21%)	16 (3.09%)	
Female age of FET, median, IQR, y	33.0 (30.0–36.0)	33.0 (30.0–36.0)	0.934
Male age of oocyte retrieval, median, IQR, y	33.0 (31.0–37.0)	34.0 (31.0–37.0)	0.148
BMI, median, IQR, kg/m^2^	22.5 (20.2–25.3)	22.3 (20.4–24.5)	0.145
Gravidity			0.002
0	543 (48.4%)	271 (52.4%)	
1	270 (24.1%)	91 (17.6%)	
≥ 2	309 (27.5%)	152 (29.4%)	
Missing	0 (0.0%)	3 (0.6%)	
Parity			0.232
0	879 (78.3%)	397 (76.8%)	
1	206 (18.4%)	93 (18.0%)	
≥ 2	35 (3.1%)	24 (4.6%)	
Missing	2 (0.2%)	3 (0.6%)	
Cause of subfertility			0.153
Female	541 (48.2%)	255 (49.3%)	
Male	128 (11.4%)	54 (10.4%)	
Mixed	414 (36.9%)	200 (38.7%)	
Unexplained	39 (3.5%)	8 (1.6%)	
Rank of oocyte retrieval			0.163
First cycle	903 (80.5%)	431 (83.4%)	
Repeated cycle	219 (19.5%)	86 (16.6%)	
Number of oocytes retrieved	11.0 (7.0–15.0)	12.0 (8.0–18.0)	< 0.001
Insemination method			0.118
IVF	646 (57.6%)	271 (52.4%)	
ICSI	471 (42.0%)	242 (46.8%)	
Both	5 (0.5%)	4 (0.8%)	
Total number of Day 5/6 blastocysts frozen, median, IQR	2.0 (1.0–3.0)	2.0 (1.0–3.0)	0.816
Source of blastocyst			< 0.001
Surplus blastocyst after fresh transfer	502 (44.7%)	160 (31.0%)	
Freeze–all strategy	620 (55.3%)	357 (69.1%)	
Rank of FET			0.001
First cycle	640 (57.0%)	339 (65.6%)	
High rank	482 (43.0%)	178 (34.4%)	
Days of estradiol treatment before progesterone administration, median, IQR	16.0 (14.0–18.0)	15.0 (14.0–18.0)	0.714
Endometrial thickness^a^, median, IQR, mm	10.0 (9.0–11.0)	10.0 (9.0–11.0)	0.018
Type of FET preparation			0.285
HRT	834 (74.33%)	397 (76.79%)	
GnRH–agonist pretreatment	288 (25.67%)	120 (23.21%)	
Blastocyst quality at vitrification^b^			0.141
Good	237 (21.12%)	93 (17.99%)	
Fair	885 (78.88%)	424 (82.01%)	
PGT cycles	193 (17.20%)	116 (22.44%)	0.012
Progesterone used for luteal support			
Intramuscular	361 (32.17%)	189 (36.56%)	0.081
Vaginal	761 (67.83%)	328 (63.44%)	

IQR interquartile range; FET frozen–thawed embryo transfer; BMI body mass index; IVF in-vitro fertilisation; ICSI intracytoplasmic sperm injection; HRT, hormone replacement treatment; PGT, preimplantation genetic testing

Data are median (IQR) or n (%), as appropriate

^a^On the first day of the progesterone administration

^b^A good-quality blastocyst as defined as an embryo≥3BB according to the grading scale by Gardner

### Primary and secondary outcomes

The LBR was significantly higher for the P6 group than for the P5 group [380/1,122(33.87%) versus 129/517 (24.95%); *P* < 0.001] (Table 2). After adjusting confoundings, the P6 regimen was associated with a significantly increase in LBR compared to the P5 regimen (adjusted odds ratio [aOR] 1.72, 95% confidence interval [CI] 1.32–2.19, *P* < 0.001).

The likelihood of biochemical pregnancy (51.96% vs. 37.72%, aOR 2.00, 95% CI 1.65–2.61) and clinical pregnancy (46.52% vs. 33.66%, aOR 1.95, 95% CI 1.55–2.46) were also significantly increased in P6 group compared with P5 group in unadjusted and adjusted GEE models. However, no significant differences were observed between the two progesterone supplementation regimens in terms of miscarriage, ectopic pregnancy or still birth rates (All *P* > 0.05).

Regarding neonatal outcomes (assessed in singleton live births), mean birth weight (3.33 ± 0.53 kg in P6 group vs. 3.23 ± 0.51 kg in P5 group, adjusted β = 0.10 kg; 95% CI: − 0.02 to 0.24), gestational age (38.28 ± 1.81 weeks vs. 38.02 ± 1.70 weeks, adjusted β = 0.33 weeks; 95% CI: − 0.10 to 0.77), and the risks of low birth weight (4.76% vs. 7.09%, adjusted OR = 0.61; 95% CI: 0.24–1.57) and preterm delivery (8.73% vs. 13.39%, aOR = 0.62; 95% CI: 0.31–1.25) showed no statistically significant differences between the two groups (Table 2).

**Table 2:** Crude and adjusted ORs of pregnancy and neonatal outcomes

	Group P6 (*N* = 1122)	Group P5 (*N* = 517)	Crude β (95% CI) /OR (95% CI)	*P*-value	Adjusted β (95% CI) /OR (95% CI)	*P*-value
Live birth	380 (33.87)	129 (24.95)	1.54 (1.22, 1.95)	0.0003	1.72 (1.32, 2.19)	0.0002
Singleton live birth	378 (33.69)	127 (24.56)	1.56 (1.23, 1.97)	< 0.001	1.73 (1.33, 2.25)	< 0.001
Biochemical pregnancy	583 (51.96)	195 (37.72)	1.79 (1.44, 2.21)	< 0.001	2.10 (1.64, 2.62)	< 0.001
Clinical pregnancy	522 (46.52)	174 (33.66)	1.71 (1.38, 2.13)	< 0.001	1.94 (1.50, 2.47)	< 0.001
Miscarriage^a^	122 (23.37)	43 (24.71)	0.93 (0.62, 1.39)	0.719	0.93 (0.61, 1.45)	0.787
Ectopic pregnancy^b^	7 (1.34)	1 (0.57)	2.35 (0.29, 19.24)	0.425	1.32 (0.14, 12.22)	0.820
Still birth^c^	13 (2.49)	1 (0.57)	4.42 (0.57, 34.00)	0.154	5.50 (0.67, 44.42)	0.110
Gestational age at delivery (weeks)^d^	38.28 ± 1.81	38.02 ± 1.70	0.26 (-0.10, 0.62)	0.154	0.33 (-0.10, 0.77)	0.144
Preterm birth (< 37 WG)^d^	33 (8.73)	17 (13.39)	0.62 (0.33, 1.15)	0.131	0.62 (0.31, 1.25)	0.203
Birth weight (kg)^d^	3.33 ± 0.53	3.23 ± 0.51	0.10 (-0.00, 0.21)	0.055	0.10 (-0.02, 0.24)	0.098
Low birth weight (< 2.5 kg)^d^	18 (4.76)	9 (7.09)	0.66 (0.29, 1.50)	0.317	0.61 (0.24, 1.57)	0.287
Large birth weight (≥ 4.0 kg)^d^	32 (8.47)	8 (6.30)	1.38 (0.62, 3.07)	0.436	1.32 (0.51, 3.49)	0.565

OR, odds ratio; WG, weeks of gestation. Data are mean ± SD or n (%), as appropriate

^a^Number of miscarriages/numbers of clinical pregnancies

^b^Number of ectopic pregnancies/numbers of clinical pregnancies

^c^Including one infant death at delivery in group P6

^d^Calculated based on the total number of singleton live births

Analysis adjusted for female age at retrieval, BMI, gravidity, PGT status, numbers of oocyte retrieval and blastocysts frozen, blastocyst origin (surplus from fresh cycle vs. freeze-all cycle), blastocyst quality, duration of estradiol treatment, endometrial thickness, FET cycle rank and progesterone used for luteal phase support

Sensitivity analyses restricted to either first FET cycles or good-quality blastocysts yielded consistent results (Supplementary Table S1) Subgroup analysis by blastocyst ploidy status revealed that extending progesterone by one additional day significantly increased LBR in non-PGT cycles (aOR 1.87, 95% CI 1.40–2.54, *P* < 0.001). However, no significant benefit was observed in cycles involving euploid D6 blastocyst transfer (aOR 1.35, 95% CI 0.80–2.20, *P* = 0.278) (Supplementary Table S2).

## Discussion

### Principal findings

This multi-center retrospective study of 1,639 single vitrified–warmed D6 blastocyst transfers under HRT revealed that extending progesterone supplementation prior to FET to 6 days was associated with a significantly higher LBR compared to the conventional 5–day progesterone regimen. Consistent with the LBR findings, both the biochemical and clinical pregnancy rates were also significantly improved in the P6 group. However, neonatal outcomes, including birth weight and gestational age at delivery, were not significantly influenced by progesterone exposure duration. These findings suggest that delayed–developing blastocysts may require extended progesterone exposure to achieve optimal endometrial synchrony in HRT–FET [20, 23].

### Strengths and limitations

A key strength of the current study is its large sample size, focusing specifically on single D6 vitrified-warmed blastocyst transfer cycles, and its dedicated investigation of the association between the duration of progesterone exposure prior to FET and live birth outcomes in HRT cycles. Moreover, the cycles were stratified into two subgroups based on ploidy status. In the subgroup with unknown embryo ploidy (non–PGT), the results were consistent with the primary findings, favoring the prolonged P6 regimen. The absence of a statistically significant improvement in LBR with prolonged progesterone exposure in euploid day-6 blastocyst transfers should be interpreted cautiously, particularly given the limited sample size in PGT subgroup. Although our data do not demonstrate a definitive LBR benefit, they do not exclude a potential advantage for chromosomally normal embryos. Notably, both biochemical and clinical pregnancy rates were significantly higher with the P6 regimen, suggesting a biologically meaningful effect that may translate into improved live birth outcomes in a larger, adequately powered study. Finally, in contrast to previous studies that were limited to either vaginal [11] or intramuscular [24] progesterone administration alone, this multicenter analysis included both routes of administration, improving the generalizability of the findings across various endometrial preparation protocols.

The study also has several limitations that need to be discussed. First, its retrospective design inherently carries risks of selection and confounding biases. Although all centers followed the Gardner scoring system, given the subjective nature of embryo grading by multiple embryologists across three clinics, variability between observers and across clinics may have introduced bias. However, the multivariable GEE analysis performed to adjust for known confounders as well as the sensitivity analyses restricted to good–quality blastocysts did not markedly modify the findings, nor the conclusions drawn. Second, key post-thaw embryo characteristics, such as re-expansion grade, hatching status, and the interval between thawing and transfer were not routinely documented across the participating centers and therefore could not be incorporated into our analyses. Furthermore, serum progesterone levels on the day of FET were not routinely measured during the study period, which represents a notable limitation. As systemic progesterone concentrations vary significantly by route of administration, the possibility of suboptimal luteal support in some patients cannot be excluded. Although the distribution of progesterone regimens (vaginal vs. intramuscular) did not differ significantly between the P5 and P6 groups (*P* = 0.081), inter- and intra-individual variability in absorption, time to steady-state concentrations, and actual endometrial exposure were not assessed [25]. Nevertheless, the varies progesterone administrations between groups likely mitigates potential bias. Finally, while the data support the use of extended progesterone priming in HRT-FET cycles involving day-6 blastocysts, it remains uncertain whether these findings extend to natural-cycle FET or to populations outside of China.

Embryos cultured in vitro typically reach the blastocyst stage by day 5 after fertilization; however, a subset develops more slowly and achieves blastulation on day 6 or later [26]. D6 blastocysts represent a distinct biological category, consistently demonstrating lower implantation and live birth rates compared with D5 blastocysts, even after adjustment for morphology and ploidy status. A leading hypothesis attributes this reduced potential, at least in part, to embryo–endometrial asynchrony when D6 blastocysts are transferred following only 5 days of progesterone exposure, a protocol originally optimized for faster-developing D5 embryos. Given that progesterone is the primary hormonal regulator of endometrial receptivity and critically defines the timing of the window of implantation [27], it is biologically plausible that D6 blastocysts may benefit from an additional day of endometrial priming to achieve better synchrony with the endometrium. Our findings are consistent with this concept and align with emerging evidence supporting extended progesterone regimens (*P* + 6) for D6 transfers. Shapiro et al. [28] first proposed individualizing progesterone exposure based on blastocyst developmental pace, and Roelens et al. [11] subsequently reported improved pregnancy outcomes with a 6-day regimen in D6 transfers, although their subgroup analyses were limited by sample size.

To date, only three randomized controlled trials have specifically investigated the impact of progesterone priming duration in HRT-FET cycles; of these, one has been published only as an abstract and another was conducted in oocyte donation cycles [9, 29, 30]. Neither trial found statistically significant differences in clinical outcomes, but both included mixed cohorts of D5 and D6 blastocysts and were underpowered to detect effects specific to D6 embryos. Similarly, a 2023 ESHRE oral presentation noted a non-significant trend toward higher live birth rates with 6 days of progesterone in HRT-FET, though the number of D6 transfers under the 5-day regimen was small [31].

In this large retrospective cohort study focusing exclusively on single vitrified–thawed D6 blastocyst transfers, we observed that a 6-day progesterone priming protocol was associated with higher live birth and clinical pregnancy rates compared with the conventional 5-day regimen. Although no statistically significant differences were observed in neonatal outcomes, the study was not powered to detect modest but clinically relevant effects on birth weight or gestational age. The observed trends toward lower rates of low birth weight and preterm delivery with the P6 regimen warrant confirmation in larger studies or meta-analyses focused on perinatal safety. While these results are encouraging and biologically coherent, randomized controlled trials are needed to confirm whether tailoring progesterone duration to blastocyst developmental day, particularly for slower-growing D6 embryos can meaningfully improve reproductive outcomes.

## Conclusion

In this retrospective cohort study of single vitrified–thawed Day 6 blastocyst transfers, a 6-day progesterone priming regimen was associated with higher live birth and clinical pregnancy rates compared to the conventional 5-day protocol. However, due to the observational nature of the data, large-scale randomized controlled trials are needed to confirm whether tailoring the duration of progesterone exposure to match the developmental timeline of slower-growing embryos can optimize reproductive outcomes. 

## Supplementary Information

Below is the link to the electronic supplementary material.

Supplementary Material 1

Supplementary Material 2

## Data Availability

Data will be made available on request.

## Abbreviations

FET: Frozen embryo transfer
LBR: Live birth rate
HRT: Hormone replacement therapy
GEE: Generalized estimating equations
PGT: Preimplantation genetic testing
WG: Weeks of gestation
IQR: Interquartile ranges
OR: Odds ratios
CI: Confidence interval
BMI: Body mass index

## References

[1] de Neubourg D, Smeenk J, Cuevas I, Rezabek K, Wingfield M, Schilling C, et al. O-159 assisted reproductive technology (ART) in Europe 2022: preliminary results generated from European registers by the ESHRE EIM consortium. Human Reproduction [Internet]. 2025;40:deaf097.

[2] Glujovsky D, Pesce R, Fiszbajn G, Sueldo C, Ciapponi A. Endometrial preparation for women undergoing embryo transfer with frozen embryos or embryos derived from donor oocytes. Cochrane Database Syst Rev. 2007;65:CD006359. 10.1002/14651858.CD006359.pub2 20091592

[3] Zaat TR, Kostova EB, P ShowellF MolM, van Wely KG. Obstetric and neonatal outcomes after natural versus artificial cycle frozen embryo transfer and the role of luteal phase support: a systematic review and meta-analysis. Hum Reprod Update. 2023;29:634–54. 37172270 10.1093/humupd/dmad011 PMC10477943

[4] Liu X, Li W, Wen W, Wang T, Wang T, Sun T, et al. Natural cycle versus hormone replacement therapy as endometrial Preparation in ovulatory women undergoing frozen-thawed embryo transfer: the compete open-label randomized controlled trial. PLoS Med [Internet] Public Libr Sci. 2025;22:e1004630. 10.1371/journal.pmed.1004630 PMC12193059 40561125

[5] Marques LM, Muylder A, Van, Brijs M, Lawrenz B, Fatemi HM, Luyten J. Cost-effectiveness of single euploid frozen embryo transfer in a true natural versus a hormonal replacement cycle. Fertil Steril. 2025. 10.1016/j.fertnstert.2025.06.030 40562298

[6] Roelens C, Blockeel C. Impact of different endometrial Preparation protocols before frozen embryo transfer on pregnancy outcomes: a review. Fertil Steril [Internet]. 2022;118:820–7. 36273850 10.1016/j.fertnstert.2022.09.003

[7] Pirtea P, Toner JP, de Scott RT. Let’s not abandon programmed frozen embryo transfers yet: a countercurrent perspective. Reprod Biomed Online. 2024;49:104365–104365. 39243633 10.1016/j.rbmo.2024.104365

[8] Mackens S, van Santos-Ribeiro S, et al. Frozen embryo transfer: a review on the optimal endometrial Preparation and timing. Hum Reprod. 2017. 10.1093/humrep/dex285 29025055

[9] Van de VA, Polyzos NP, Van LL, Mackens S, Stoop D, Camus M, What is the optimal duration of progesterone administration before transferring a vitrified-warmed cleavage stage embryo? a randomized controlled trial. Hum Reprod. 2016. 10.1093/humrep/dew045 27005893

[10] Kaye L, Will EA, Bartolucci A, Nulsen J, Engmann L. Pregnancy rates for single embryo transfer (SET) of day 5 and day 6 blastocysts after cryopreservation by vitrification and slow freeze. J Assist Reprod Genet. 2017;34:1–7. 10.1007/s10815-017-0940-4 PMC5476550 28500451

[11] Roelens C, Santos-Ribeiro S, Becu L, Mackens S, Van Landuyt L, Racca A, et al. Frozen-warmed blastocyst transfer after 6 or 7 days of progesterone administration: impact on live birth rate in hormone replacement therapy cycles. Fertil Steril [Internet]. 2020;114:125–32. 32553469 10.1016/j.fertnstert.2020.03.017

[12] Wen W, Zhang D, Liu X, Shi J, Cai H. Embryo development and live birth in women with one previously failed full IVF/ICSI cycle. J Assist Reprod Genet. 2024;41. 10.1007/s10815-024-03107-8 PMC11224215 38739214

[13] Shi W, Jin L, Liu J, Zhang C, Mi Y, Shi J, et al. Blastocyst morphology is associated with the incidence of monozygotic twinning in assisted reproductive technology. Am J Obstet Gynecol [Internet]. 2021;225:654.e1-654.e16. 10.1016/j.ajog.2021.06.101 34245681

[14] Gardner DK, Lane M, Stevens J, Schlenker T, Schoolcraft WB. Blastocyst score affects implantation and pregnancy outcome: towards a single blastocyst transfer. Fertil Steril [Internet]. 2000;73:1155–8. 10856474 10.1016/s0015-0282(00)00518-5

[15] Shi W, Xue X, Zhang S, Zhao W, Liu S, Zhou H, et al. Perinatal and neonatal outcomes of 494 babies delivered from 972 vitrified embryo transfers. Fertil Steril [Internet]. 2012;97:1338–42. 22464083 10.1016/j.fertnstert.2012.02.051

[16] He T, Xue X, Shi J. Embryo retention and live birth in frozen embryo transfer cycles: a cohort study. Fertil Steril. 2025;123. 10.1016/j.fertnstert.2024.09.035 39322102

[17] Pan D, Yang J, Zhang N, Wang L, Li N, Shi J, et al. Gonadotropin-releasing hormone agonist downregulation combined with hormone replacement therapy improves the reproductive outcome in frozen–thawed embryo transfer cycles for patients of advanced reproductive age with idiopathic recurrent implantation failure. Reproductive Biology Endocrinol [Internet]. 2022;20:26. 10.1186/s12958-022-00897-3 PMC8812179 35115007

[18] Zegers-Hochschild, Fernando, Adamson G, David D, et al. The international glossary on infertility and fertility Care, 2017 - ScienceDirect. Fertil Steril. 2017;108:393–406. 28760517 10.1016/j.fertnstert.2017.06.005

[19] Vitagliano A, Paffoni A, Viganò P. Does maternal age affect assisted reproduction technology success rates after euploid embryo transfer? A systematic review and meta-analysis. Fertil Steril [Internet]. 2023;120:251–65. 36878347 10.1016/j.fertnstert.2023.02.036

[20] Haley G, Alonso MC, Erkan K, Human F, Baris A, Juan GV, et al. Does endometrial thickness impact live birth rate following a frozen embryo transfer: outcomes of 30676 euploid single embryo transfers. Hum Reprod. 2025. 10.1093/humrep/deaf129 40639807

[21] Genovese H, Mayo CA, Kalafat E, Fatemi H, Ata B, Garcia-Velasco J, et al. Does endometrial thickness impact live birth rate following a frozen embryo transfer: outcomes of 30 676 euploid single embryo transfers. Human Reproduction [Internet]. 2025;deaf129. 10.1093/humrep/deaf129 40639807

[22] Chloé M, Timon J, Mathilde B, Léa M, Louis M, Christelle LM, et al. High serum estradiol levels on the day of frozen blastocyst transfer are associated with increased early miscarriage rates in artificial cycles using transdermal estrogens. Hum Reprod. 2025;5. 10.1093/humrep/deaf037 40052481

[23] Franasiak JM, Ruiz-Alonso M, Scott RT, Simón C. Both slowly developing embryos and a variable Pace of luteal endometrial progression May conspire to prevent normal birth in spite of a capable embryo. Fertil Steril [Internet]. 2016;105:861–6. 26940791 10.1016/j.fertnstert.2016.02.030

[24] Bhoi N, Yarali H, Murdia K, Murdia N, Chandra V, Suwalka I, et al. Comparison of live birth rates following the transfer of day-6 blastocysts on the 6th versus 7th day of progesterone exposure in hormone replacement treatment–frozen embryo transfer cycles. Clin Exp Reprod Med [Internet] Korean Soc Reproductive Med. 2024;52:125–33. 10.5653/cerm.2023.06527 PMC12149863 40468815

[25] Devine K, Richter KS, Jahandideh S, Widra EA, McKeeby JL. Intramuscular progesterone optimizes live birth from programmed frozen embryo transfer: a randomized clinical trial. Fertil Steril [Internet]. 2021;116:633–43. 33992421 10.1016/j.fertnstert.2021.04.013

[26] Richter KS, Shipley SK, McVearry I, Tucker MJ, Widra EA. Cryopreserved embryo transfers suggest that endometrial receptivity May contribute to reduced success rates of later developing embryos. Fertil Steril [Internet]. 2006;86:862–6. 16935284 10.1016/j.fertnstert.2006.02.114

[27] Lessey BA, Young SL. What exactly is endometrial receptivity? Fertil steril [Internet]. Elsevier. 2019;111:611–7. 10.1016/j.fertnstert.2019.02.009 30929718

[28] Shapiro BS, Daneshmand ST, Garner FC, Aguirre M, Ross R. Contrasting patterns in in vitro fertilization pregnancy rates among fresh autologous, fresh oocyte donor, and cryopreserved cycles with the use of day 5 or day 6 blastocysts May reflect differences in embryo-endometrium synchrony. Fertil Steril [Internet]. 2008;89:20–6. 17224151 10.1016/j.fertnstert.2006.08.092

[29] Ding J, Rana N, Dmowski WP. Length of progesterone treatment before transfer and implantation rates of frozen-thawed blastocysts. Fertil Steril [Internet]. 2007;88:S330–1.

[30] Blázquez A, Miguel-Escalada I, Portas-Gómez L, Guillén-Quílez JJ, Mataró D, Popovic M et al. Prolonged vaginal progesterone supplementation does not improve outcomes for Day 6 frozen blastocyst transfers in oocyte donation cycles. Human Reproduction [Internet]. 2025;deaf205. 10.1093/humrep/deaf205 41206359

[31] Boel Povlsen B, Alsbjerg B, Humaidan P. O-024 Exploring the optimal transfer day of day 6 (D6) vitrified blastocysts in Hormone Replacement Therapy Frozen Embryo Transfer (HRT-FET) cycles - a cohort study. Human Reproduction [Internet]. 2023;38:dead093.024.

