# Supplementary material for: Duration of progesterone exposure before frozen embryo transfer impacts live birth rates following single vitrified-thawed day 6 blastocyst transfer: a multicenter cohort study
Source: Contracept Reprod Med. 2026 Jan 28;11:14. doi: 10.1186/s40834-026-00425-3 (PMC12924586; doi:10.1186/s40834-026-00425-3)
Supplement: Supplementary file 1 — Supplementary Material 1 [file 40834_2026_425_MOESM1_ESM.docx]

Supplementary table 1. Sensitive analyzes

| Live birth rates | Group P6 | Group P5 | OR (95% CI) | *P*-value | Adjusted OR (95% CI) | *P*-value |
| --- | --- | --- | --- | --- | --- | --- |
|  | (%) n/N | (%) n/N |  |  |  |  |
| First FET cycle^a^ | (26.84%) 91/339 | (36.09%) 231/640 | 1.54 (1.15, 2.06) | 0.004 | 1.61 (1.19, 2.23) | 0.002 |
| Good quality of blastocysts transfer ^b^ | (48.10%) 114/237 | (29.03%) 27/93 | 2.27 (1.35, 3.79) | 0.002 | 2.40 (1.39, 4.27) | 0.002 |

OR, odds ratio; PGT, Pre-implantation genetic test. Data are (%), n/N

^a^ Analysis adjusted for female age at retrieval, BMI, gravidity, PGT status, numbers of oocyte retrieval and blastocysts frozen, blastocyst origin (surplus from fresh cycle vs. freeze-all cycle), blastocyst quality, duration of estradiol treatment, endometrial thicknes and progesterone used for luteal phase support.

^b^Analysis adjusted for female age at retrieval, BMI, gravidity, PGT status, numbers of oocyte retrieval and blastocysts frozen, blastocyst origin (surplus from fresh cycle vs. freeze-all cycle), duration of estradiol treatment, endometrial thickness, FET cycle rank and progesterone used for luteal phase support.
