# Supplementary material for: Duration of progesterone exposure before frozen embryo transfer impacts live birth rates following single vitrified-thawed day 6 blastocyst transfer: a multicenter cohort study
Source: Contracept Reprod Med. 2026 Jan 28;11:14. doi: 10.1186/s40834-026-00425-3 (PMC12924586; doi:10.1186/s40834-026-00425-3)
Supplement: Supplementary file 2 — Supplementary Material 2 [file 40834_2026_425_MOESM2_ESM.docx]

Supplementary table 2. Live birth and pregnancy outcomes between the two groups stratified by PGT status

|  | Group P6 | Group P5 | OR (95% CI) | *P*-value | Adjusted OR (95% CI) | *P*-value |
| --- | --- | --- | --- | --- | --- | --- |
| PGT cycles | N=193 | N=116 |  |  |  |  |
| Live birth | 100 (51.81) | 50 (43.10) | 1.42 (0.89, 2.26) | 0.139 | 1.35 (0.80, 2.20) | 0.278 |
| Biochemical pregnancy | 132 (68.39) | 56 (48.28) | 2.32 (1.44, 3.72) | 0.005 | 2.30 (1.36, 3.83) | 0.019 |
| Clinical pregnancy | 125 (64.77) | 56 (48.28) | 1.97 (1.23, 3.15) | 0.001 | 1.90 (1.24, 3.14) | 0.004 |
|  |  |  |  |  |  |  |
| Non-PGT cycles | N=929 | N=401 |  |  |  |  |
| Live birth | 280 (30.14) | 79 (19.70) | 1.76 (1.32, 2.33) | <0.001 | 1.87 (1.40, 2.54) | <0.001 |
| Biochemical pregnancy | 451 (48.55) | 118 (29.43) | 1.78 (1.40, 2.27) | <0.001 | 2.00 (1.53, 2.62) | <0.001 |
| Clinical pregnancy | 397 (42.73) | 139 (34.66) | 1.79 (1.39, 2.30) | <0.001 | 1.86 (1.50, 2.58) | <0.001 |

OR, odds ratio; PGT, Preimplantation genetic test. Data are n (%)

Analysis adjusted for female age at retrieval, BMI, gravidity, numbers of oocyte retrieval and blastocysts frozen, blastocyst origin (surplus from fresh cycle vs. freeze-all cycle), blastocyst quality, duration of estradiol treatment, endometrial thickness, FET cycle rank and progesterone used for luteal phase support.
